# The effect of anaerobic digestate derived composts on the metabolite composition and thermal behaviour of rosemary

**DOI:** 10.1038/s41598-019-42725-6

**Published:** 2019-04-24

**Authors:** M. A. Bustamante, I. Nogués, S. Jones, G. G. Allison

**Affiliations:** 1 Department of Agrochemistry and Environment, Miguel Hernandez University, EPS-Orihuela, ctra. Beniel Km 3.2, 03312 Orihuela (Alicante), Spain; 2 Consiglio Nazionale delle Ricerche, Istituto di Biologia Agroambientale e Forestale, Via Salaria Km. 29, 300–00016 Monterotondo Scalo, Roma Italy; 3 Institute of Biological, Environmental & Rural Sciences (IBERS), Aberystwyth University Gogerddan, Aberystwyth, Ceredigion SY23 3EE UK

**Keywords:** Plant sciences, Plant physiology

## Abstract

The study reports on the effect of anaerobic digestate derived composts on the metabolite composition and thermal behaviour of rosemary (*Rosmarinus officinalis* L.). Plants were cultivated in semiarid soil under four different fertiliser treatments (composts of anaerobic digested cattle (C) or pig slurry (P) at 30t/ha and 60 t/ha, and two control treatments (inorganic fertiliser and no fertiliser application). Samples of leaves and stems were analysed to investigate the effect of treatment on chemical composition and thermochemical properties. Three orthogonal analytical approaches were used, namely: Fourier transform mid infrared spectroscopy (FTIR), gas chromatography/mass spectrometry (GC/MS) and thermochemical gravimetric analysis (TGA). FTIR and GC/MS showed fertiliser treatment resulted in tissue specific changes in sample metabolite composition. Fertiliser treatment was detected to change the thermogravimetric properties of the leaf samples and from inorganic and composted pig slurry digestate treatments had greater ash content and lower proportions of fixed carbon compared with samples from the unfertilised control treatment. This study provides information on how the composition of rosemary might be altered by fertiliser application in regions of poor soil, and has implications for biomass quality when rosemary is grown on semi-wild sites for the purpose of soil improvement.

## Introduction

Rosemary (*Rosmarinus officinalis* L., Lamiaceae) is an aromatic shrub native to the Mediterranean region, and it is grown as a common herb around the world for culinary use. The species also has economic importance and is cultivated widely for essential oil production^1^. The essential oils of *Rosmarinus officinalis* are used in a diverse range of industry sectors including the food, cosmetic and pharmaceutical sectors, and there is considerable interest in their health related e.g. antioxidant, antibacterial, antifungal and anti-inflammatory properties^2,3^. Rosemary also has a role in soil rehabilitation in Mediterranean regions where it is grown to alleviate soil erosion. Since it is drought-tolerant it grows on soils ranging from rocky to sandy, providing sites have adequate drainage and a minimum soil depth of about 0.2 m^4,5^. Furthermore, rosemary can be planted for the phytostabilisation of soils contaminated with heavy metals^6–9^ and its growth and oil composition are relatively unaffected by growth on heavy-metal contaminated soils^4^.

Several studies have investigated the effect of growth conditions on rosemary yield and essential oil composition^10–12^ and their results implicate fertiliser application and dose as important agronomic factors. However, very few studies have been reported of the effect of fertiliser type and application rate on the characteristics of rosemary plants^13,14^ and these studies mainly focused on yield and oil quality. Several groups have reported that the use of fertilisers and/or amendments may alter vegetal cell wall content and composition^15,16^, but there is little or no knowledge of the effect of organic fertilisers on the composition of rosemary. Primary and secondary metabolites are the intermediate or ultimate products of complex networks of biochemical pathways^17^, whose functioning depends on substrates availability and/or enzyme activities, which at the same time are highly dependent on micro and macronutrient availability. As organic fertilizers are sustained sources of nutrients due to slow release during decomposition, soil nutrients are more available to plants over the time. Changes in macronutrient availability (e.g. N, P and K) indeed has a direct effect on metabolism, since macronutrients are directly involved in essential cellular processes, and in many cases the biosynthesis of important organic molecules e.g. proteins, nucleic acids, co-enzymes, phytohormones and secondary metabolites. Even elements that at first inspection would appear to be required in only very small amounts i.e. the micronutrients e.g. Mn, Fe, Zn and Cu, can have major influence on tissue composition and metabolism due to their requirement as cofactors for enzyme activity.

The methods used in this study all provide rich data on different aspects of sample composition, and are therefore orthogonal and complementary in nature. Gas chromatography coupled with mass spectrometry (GC/MS) is a fast and accurate technique widely used to characterise a great variety of biological and chemical samples. The strength of the technique lies in the combination of exceptional chromatographic resolution of chemical components in the sample coupled to mass spectral information that directly reflects molecular structure. The robustness and reproducibility of GC/MS has favoured adoption of GC/MS for many metabolomics studies, in which complex multivariate chemical relationships between samples are detected by multivariate data analysis^18,19^. Fourier transform infrared spectroscopy (FTIR) also provides complex chemical data on sample composition, and mid-IR spectra informs of chemical bonds present in pure samples. Whilst it is possible to quantify primary and secondary metabolite concentrations in samples and tissues using regression models {Allison, 2011 #57;Schulz, 2005 #41;Allison, 2009 #2;Allison, 2009 56}. Lastly, we have used thermogravimetric analysis (TGA) to provide information on mass-fraction i.e. moisture, volatile and fixed carbon content as samples are heated in the absence of oxygen. TGA has been used to characterise organic wastes and composts^20,21^, soils^22,23^, lignocellulose composition and energy grass feedstock quality^15^, and the technique provides valuable information on the kinetics of thermal decomposition^24^. We have used these methods to provide orthogonal, chemically meaningful data for chemometric analyses to visualise chemical/compositional relationships between samples and thereby investigate the effect of fertiliser dose and type on rosemary plants in a global context. This study aims to increase our understanding of the likely compositional effects of organic fertiliser application on rosemary crops and on populations grown in semi-wild regions for the purpose of soil stabilisation.

## Materials and Methods

### Characteristics of the soil and organic amendments

The soil used in this study was obtained from the surface layer (0–20 cm) of a semiarid agricultural area, abandoned for a period of ten years in Montelibretti, a town in the Metropolitan City of Rome, in Lazio (Italy). Before being abandoned, the area had been under intensive agriculture for more than 20 years. The soil was air dried before being passed through a 2 mm sieve. The soil from this site is a highly calcareous loam soil, that is slightly alkaline (pH = 7.6), and has low salinity (0.10 dS/m) and poor total organic C content (0.75% C).

The composts used in this study comprised of solid fractions from the anaerobic digestion of cattle and pig slurry (75% by dry mass of digested cattle slurry (compost C), or digested pig slurry (compost P) respectively) mixed with 25% by dry mass of vine shoot prunings. The anaerobic cattle and pig digestates used were obtained after a continuous, mesophilic anaerobic digestion of 4.3% cattle manure and 11.6% maize oat silage (fresh mass basis) and of pig slurry, respectively. Compost C was prepared at semi-industrial scale (1800 kg) by the Rutgers static pile composting system, while compost P was prepared in a thermo-composter (150 kg) by the turning composting system. During composting, both mixtures reached temperature values higher than 55 °C, which guarantees the sanitization of the composts. The complete description of the composting process is detailed elsewhere^25,26^. Chemical analysis indicated that both composts were very suitable for their agricultural use (Table 1): the ratio of total organic carbon (TOC) to total nitrogen (C/N) in both composts was below the value of 20^25^ and the contents of N, P and K were similar to those found in composts derived from livestock manure. In addition, in both composts the cation exchange capacity (CEC) (>60 meq/100 g organic matter) and the germination index (GI) (>60%) fulfilled the limits established for mature composts^27^.

**Table 1:** Main characteristics of the cattle (C) and pig (P) anaerobic digestate derived composts.

Parameter	C	P
C/N	11.9	11.4
Total N (g/kg)	29	30.3
P (g/kg)	6.95	33.7
K (g/kg)	16.4	9.78
CEC (meq/100 g)	124	171
CEC/TOC	2.47	3.36
GI (%)	99.6	79.8

CEC: cation exchange capacity; TOC: total organic carbon; GI: germination index.

### Experimental design

Polyethylene pots were filled with 1 kg of soil mixed thoroughly with composts P or C at two doses (expressed on a fresh mass basis): a) Low dose (L) comprised 11.54 g compost per kg soil (corresponding to a dose of 30 t/ha) and b) high dose (H) comprised 23.08 g of compost per kg soil (equivalent to a dose of 60 t/ha). Positive and negative control treatments were included in the experimental design: The positive treatment (InOrg) being an inorganic fertilisation application of NPK in the proportion of 100:60:73. This was obtained by adding 192 mg kg^−1^ soil of the commercial fertiliser Nitrophoska top 20 (NPK = 20:5:10), and 26 mg kg^−1^ of Monopotasic phosphate (NPK = 0:52:34). The negative control treatment (Control) comprised soil without the addition of fertiliser. The NPK ratios of the amended soils were comparable with soils fertilised with inorganic fertiliser (NPK of 100:60:73). Each treatment was replicated six times.

Genetically identical rooted cuttings of rosemary were planted, one in each of the pots filled with the corresponding soil treatment. Fifty cuttings had been prepared for this purpose from a single parent plant. The cuttings were each approximately 5 cm long and immediately after removal from the parent they were stripped of their lower leaves, dipped into a hormone-containing rooting powder, planted in rooting pots with peat, and maintained in a greenhouse for a period of two months whist rooting took place. After this period, plants of similar length were selected and carefully re-potted in the treatment pots, which were distributed in a randomised complete block design inside a heated greenhouse. The cutting roots were carefully washed with distilled water to remove any particles attached before being planted. All of the pots (n = 36) were maintained for six months at a controlled temperature of 25 °C. Watering of the pots was monitored gravimetrically and maintained at 50% of field capacity throughout the experiment. After six months, the plant foliar tissues were collected, washed with deionised water to remove any attached particles, separated into leaves and stems, and dried in an air-forced oven at 60 °C for 72 h. The dried samples were then ground to a mean size of 0.5 mm for later analyses.

### Analysis by gas chromatography/mass spectrometry (GC/MS)

Samples were prepared for analysis by GC/MS by derivatisation to the methoxime/trimethylsilyl (MOX-TMS) derivative according to the modified method described by Parveen *et al*.^28^. Portions of each ground sample (60 mg) were extracted into 500 µL of ethanol at 40 °C in a screw top microcentrifuge tube, into which was also placed a stainless steel pellet. The tubes were, shaken for 5 min in a Retsch ball mill at 30 impacts s^−1^, after which 500 µL of chloroform was added to each tube and the shaking repeated. Following the second shaking, the tubes were shaken for a final time after addition of 400 µL of water. The tubes were then subjected to centrifugation for 5 min at 13000 g in a benchtop centrifuge to separate the phases. A portion (50 µL) of the upper (aqueous) phase from each sample was transferred to an 11 mm glass GC vial (Chromocol) and the contents dried under a flow of oxygen free nitrogen in a hot block at 40 °C. Samples were derivatised in a two-step procedure: Firstly 30 µL of freshly prepared methoxyamine hydrochloride (MOX) dissolved in pyridine (20 mg/mL) was added to each vial. The vials were crimp capped with Teflon sealed lids, and the sealed vials incubated in a hot block at 90 °C for 15 min. The samples were then removed from the block and after cooling they were uncapped. To each vial 20 µL of N, O-bis(trimethylsilyl)trifluoroacetamide (BSTFA) was added, the vials resealed using new caps, and then incubated for a further 15 min at 90 °C.

The derivatised samples were analysed on a GC 6890 (Agilent Technologies, Palo Alto, CA) coupled to a 5973 N mass spectrometer (Agilent Technologies, Palo Alto, CA). The GC was operated under electronic pressure control at a flow rate of 1 ml/min He carrier gas and 1 µL injections of each sample were made onto the column (30 m × 0.25 mm i.d. Rtx-5Sil MS with an integrated guard column and a 0.25 mm film (Restek GmbH, Bad Homburg, Germany) via a split/splitless capillary inlet in the split mode set to a 5:1 split ratio. Inlet temperature was maintained at 280 °C.

The temperature of the oven on injection was 80 °C. This temperature was held for 2 mins and then ramped to 300 °C at a rate of 15 °C/min, held for 2 mins and then finally raised to 330 °C at a rate of 50 °C/min and held for 5 mins to purge the column. The temperatures of the transfer line, the ion source and the quadrupole were 330 °C, 230 °C and 150 °C respectively. Mass spectra were monitored by quadrupole scanning over a range of 50 m/z to 550 m/z. Tuning and all other settings of the mass spectrometer were according to the manufacturer’s recommendations.

The data from these analyses was either converted into.csv files using Enhanced Chemstation (G1701CA, Agilent) and imported into Matlab for direct analysis of TIC data, or alternatively, the TICs were deconvoluted using AMDIS32 software (NIST) and individual mass tags identified tentatively by automated comparison with deconvoluted MSTs in a public access retention indexed mass spectral library (http://csbdb.mpimp-golm.mpg.de/) using MSSearch software (NIST). Compounds that appeared in three or fewer samples were discarded as erroneous. Peak abundances of compounds occurring as multiple peaks in the chromatogram were combined for statistical analysis.

### Analysis by Fourier transform mid-infrared spectroscopy (FTIR)

Infrared spectra were collected in duplicate from ground samples according to the method described by Allison *et al*.^29^ by FTIR from 4000 to 600 cm^−1^ using an Equinox 55 FTIR spectrometer (Bruker UK Ltd., Coventry, UK) fitted with a Golden Gate attenuated total reflection (ATR) accessory (Specac Ltd., Slough, UK). Spectra were averaged over 64 scans at a resolution of 2 cm^−1^ and corrected for background absorbance by subtraction of the spectrum of the empty ATR crystal. Spectra of soluble metabolites were also collected by analysis of the ethanolic extracts obtained in section 2.3. Small volumes (10 µl) of the extracts were spotted onto 96 well patters silicon plates (Bruker UK Ltd.) and allowed to dry at 40 °C. Spectra from the dried extract spots were measured on the HTS plate-reader accessory (Bruker UK Ltd.) of the Equinox 55 spectrophotometer as described using Opus Lab software (Bruker UK Ltd.), with position A1 of the plate being kept as a reference blank position.

FTIR spectra were converted to.csv format using Opus software (v 4.2, Bruker) and imported into Matab R2014b (version 8.4, MathWorks Inc.) using custom importation scripts and analysed using the PLS Toolbox (Eigenvector Research Inc., version 8.1.1).

### Proximate analyses by thermogravimetric (TGA) analysis

Thermo-gravimetric experiments (TGA) were carried out using a Perkin-Elmer Pyris 1 TGA analyser. The samples of leaves and stems of the rosemary plants were heated in nitrogen at a flow rate of 50 mL min^−1^ using the following 2-step temperature program. The first step consisted of the thermogravimetic analysis: (i) Heat from 40 to 105 °C at 10 °C min^−1^; (ii) hold at 105 °C for 10 min; (iii) heat from 105 to 905 °C at 10 °C min^−1^; (iv) hold at 905 °C for 15 min; and (v) cool from 905 to 105 °C at 25 °C min^−1^. The second part of the analysis was to measure the proportion of ash in the samples. This took place once the TGA section had concluded and consisted of the sample being heated to 580 °C, at a rate of 100 °C min^−1^ in the presence of air, and holding that temperature for 40 minutes. During this period the fixed carbon is lost as CO_2_ and what is left is the mass of the ash.

Proximate analysis was performed on the TGA data to calculate the relative proportions (wt. %) of moisture, volatiles, and char (ash + fixed carbon) for each sample. The moisture content was calculated from the mass loss which occurred between 40 and 105 °C, the volatiles from the mass loss between 105 and 550 °C, and the char from 550 to 900 °C. Data from TGA were converted to text files using Pyris software (Perkin Elmer) and imported into Excel (Microsoft), where the data were processed using a custom spreadsheet to calculate the proportions of moisture, volatile carbon and char.

### Statistical analysis

General statistical analysis of the experimental data was made using Genstat version 8.1 (VSN International Ltd.). All chemometric analysis of the data was made in Matab R2014b (version 8.4, MathWorks Inc.) with the PLS Toolbox (Eigenvector Research.Inc.).

### Link to experimental data

All Matlab importation scripts are available on request from the corresponding author. The experimental data is available by following the OSF link https://osf.io/pua8t/?view_only=868a599487ca4fc0a81757a3ac4c212c, where for convenience is in the form of.mat and excel files. The raw data is available from the corresponding author upon request.

## Results and Discussion

### FTIR spectroscopic analysis of ground samples

Visual inspection of the spectra from the leaf and stem samples, recorded from 4500–700 cm^−1^, allowed several of the functional spectral bands to be assigned to molecular structures using literature data^30–32^. Absorbance in the range *ca*. 1200–850 cm^−1^ has been correlated with the presence of carbohydrates, especially of cellulosic (*ca*. 1100 cm^−1^) and hemicellulosic compounds (around 815 cm^−1^ ^30–32^. Absorbance peaks between 1260–1180 cm^−1^ may be due to functional groups related to phenolic compounds^31^. Additionally, functional groups associated to components containing lignin were identified at around 1510 cm^−1^^30^. A preliminary analysis of the ATR-FTIR spectra (n = 54, see Supplementary Material Fig. S1A) from the leaf and stem samples by principal components analysis (PCA) was carried out after pre-processing of the spectra by multiplicative scatter correction, smoothing (1st order polynomial with a 15 point window) and mean centring. Model over-fitting was minimised by using a venetian blind cross validation protocol (20 PCs with 6 data splits) to indicate the optimal number of components to include in the model. Cross validation indicated a 3-component model that explained 97.43% of variance best fitted the data (root mean square error of cross validation (RMSECV) and correlation (RMSEC) of 0.00134 and 0.00107 respectively). This preliminary model of the leaf and stem samples showed clear separation of the samples by tissue type along principal component axis 1 (86.13% variance, Fig. S1B), which can be attributed to the different composition associated to the type of plant tissue. The higher scores of the stem sample spectra were due primarily to larger absorbance at 1032 cm^−1^, possibly indicating discriminatingly greater cellulose content in the stem samples, whilst the leaf samples had higher absorbance at 2924, 2851, 1680, 1541, 1441, 1391, 1281 and 1177 cm^−1^ (Fig. S1C).

**Figure 1:**
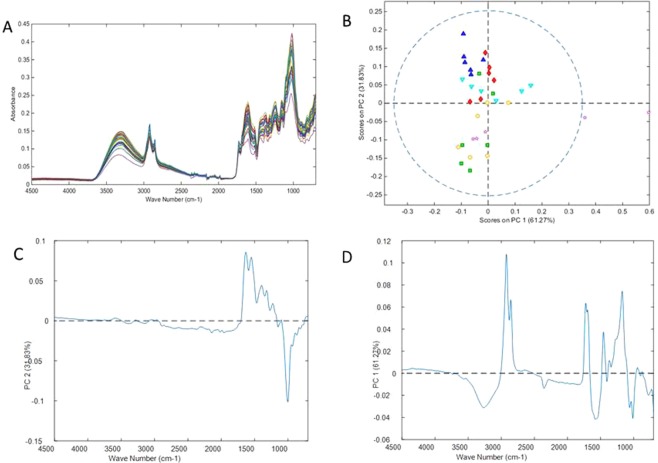
PCA of mid IR ATR spectra from ground leaf samples. (**A**) Spectra of samples. (**B**) Scores plot for PC1 vs. PC2 (red diamonds = CH; green square = CL dose; dark blue triangle PH; light blue inverted triangle = PL; pink star = control; yellow circle = InOrg; dotted line = 95% confidence level). (**C**) Loading plot for PC1. (**D**) Loading plot for PC2. CH: compost from anaerobic digested cattle slurry at dose 60 t/ha; CL: compost from anaerobic digested cattle slurry at dose 30 t/ha; PH: compost from anaerobic digested pig slurry at dose 60t/ha; PL: compost from anaerobic digested pig slurry at dose 30t/ha; InOrg: inorganic fertiliser.

Further PCA exploration was made after separation of the spectra into tissue specific data sets to better explore the variance within samples from each tissue. A 2-component PCA model of the 36 leaf sample spectra (Fig. 1A), (RMSECV and RMSEC of 0.00125 and 0.00101 respectively) explained 93.1% of the variance, with the sample scores showing some grouping according to experimental treatment along the second principal component axis (31.83% of variance). In contrast, PC1 (61.27% variance) largely explained within group variation, particularly for the control sample group of which 2 samples were obvious outliers (Fig. 1B). The loadings underlying PC axis 2 are shown in Fig. 1C. The major loadings of this component lie predominantly in the finger print region, with peaks at 1620 cm^−1^, 1539 cm^−1^ (greater contributions in both high dose composts, and the low dosage pig compost) and 993 cm^−1^ (greater contributions in both low dosage cow compost, and both control groups) suggesting separation between groups may be occurring on the basis of differing concentrations of possibly cellulose and lignin.

The loadings of PC1 are shown in Fig. 1D for completeness. Twafeeq *et al*.^14^ also reported similarity between rosemary treated with inorganic and control (without fertilisation) treatments when comparing composition and essential oil yield in rosemary plants.

Remodelling after removal of the 2 most extreme outlier samples from the data effectively removed the first component from the model, but sample grouping by treatment and model fit was not improved. Similarly, PCA using only variables 1747–725 cm^−1^ did not improve model fit or grouping (data not shown).

**Figure 2:**
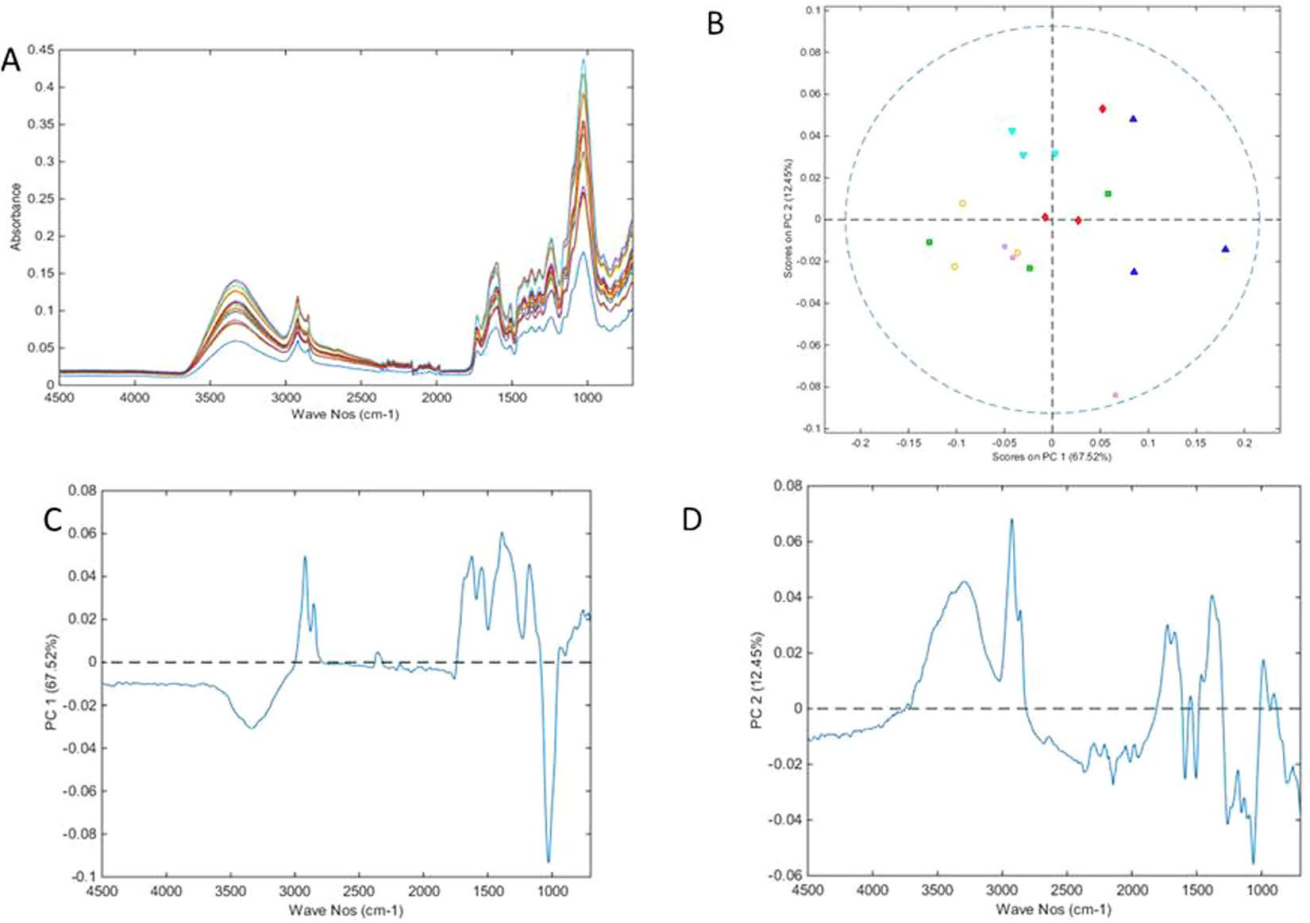
PCA of mid IR ATR spectra from ground stem samples. (**A**) Spectra of samples. (**B**) Scores plot (red diamonds = CH; green square = CL; dark blue triangle PH; light blue inverted triangle = PL; pink star = control; yellow circle = InOrg; dotted line = 95% confidence level). (**C**) Loading plot for PC1. (**D**) Loading plot for PC2. CH: compost from anaerobic digested cattle slurry at dose 60 t/ha; CL: compost from anaerobic digested cattle slurry at dose 30 t/ha; PH: compost from anaerobic digested pig slurry at dose 60t/ha; PL: compost derived anaerobic digested pig slurry at dose 30t/ha; InOrg: inorganic fertiliser.

The mid-IR spectra of the stem samples (n = 18) are shown in Fig. 2A. PCA using identical pre-processing and cross validation parameters to those used for the leaf model, PCA resulted in a 4 component model (RMSEC = 0.00058, RMSECV = 0.00107) that explained 92.14% of the experimental variance. Plotting the scores of the first component (67.52% variance) against the second (12.45% variance) allowed partial separation of sample scores according to experimental treatment (Fig. 2B), although separation was less well resolved compared to the PCA model of the leaf samples (Fig. 1B). Strong outlier samples were not detected along either principal component axes. The loadings for PC1 appear to be a combination of the PC1 and PC2 loadings from the leaf sample PCA model, with positive loadings for PC1 at 2922, 2849, 1626, 1549, 1383 and 1182 cm^−1^ and negative loadings at 3327 and 1030 cm^−1^ (Fig. 2C). Loadings for PC2 were more complex (positive loading peaks at 3292, 2926, 2866, 1728, 1666, 1383, and 990 cm^−1^, negative loading peaks at 2145, 1585, 1501, 1256 and 1057 cm^−1^, Fig. 2D). Generally, the stem samples of rosemary plants grown in the compost-derived treatment with high dose samples had more positive scores in PC1 than the low dose samples, and the non-organic fertiliser treated samples (control and InOrg) had negative scores along PC axis 2 (Fig. 2B). This result also indicates that the control and inorganic treatments had similar effects on rosemary stem sample chemical composition, and quite different from the effect observed by PCA, of treatment with organic amendments for rosemary leaves. These results are in concordance with those of Cala *et al*.^4^, who also reported differences in composition (nutrient content) in rosemary plants grown in organic amended soils compared to those grown in the soil without amendment, in a study that evaluated the effects of organic waste compost on *Rosmarinus officinalis* grown in degraded soil.

### FTIR analysis of plant ethanolic extracts

Preliminary analysis of the mid IR transmission spectra (n = 54) of soluble metabolites, collected from the ethanolic extracts from the leaf and stem samples dried onto silica plate, showed one of the stem samples to be unusual, probably a consequence of sample preparation, and this sample was removed from the spectral data set (data not shown). PCA modelling of the remaining 53 spectra (data not shown) using pre-processing and cross validation protocols identical to those described earlier for analysis of the ground samples resulted in a 4 component model that explained 96.17% of variance (RMSECV and RMSEC of 0.00447 and respectively 0.00297), which did not allow separation of the spectra according to tissue. PCA using a restricted data set extending from 1122–1598 cm^−1^ gave a 3-component model and explained 94.17% variance in the data (RMSECV and RMSEC of 0.00555 and 0.00377 respectively). However, this model again did not indicate strong grouping according to experimental treatment (data not show).

PCA models were developed separately on stem (n = 17) and leaf spectral data sets (n = 36). A 4-component PCA model explained 95.86% of sample variance in the leaf sample spectra (RMSEC and RMSECV of 0.002027 and 0.003175 respectively, see Fig. S2), whilst a 6-component model explained 99.51% of variance in the stem sample data (RMSEC and RMSECV of 0.0012 and 0.0047 respectively, see Fig. S3). In both models samples separated only partially according to treatment along PC1 (74.77% and 87.72% of variance in the leaf and stem samples respectively), and in both models treatment with high rate of pig slurry digestate compost (PH) resulted in samples having more positive scores on PC1 compared with treatment of the same compost at low dose (PL), or treatment with either composted cattle manure digestate (CH and CL). In contrast, the InOrg and control samples had negative scores on Principal Component axis 1 (Figs S2B and S3B), and furthermore, the loading for PC1 were highly similar for both models (Figs S2C and S3C), and also to the loading observed for the PCA model of the entire spectral data. It would therefore appear that in broad terms, there is considerable similarity between the ethanol extractable metabolome from stems and leaves with these samples.

The grouping of the samples into treatment groups by PCA of spectra data from both ground and ethanol extracted samples indicate a stronger effect of treatment by both type and application rate (in the treatment with composted pig slurry digestate), and a clear differentiation between organic and inorganic fertilising treatments. Some authors have reported that the sensitivity of ATR-FTIR to detect differences in sample chemistry is lower when is used directly on plant tissues, as compared with analysis of sample extracts, or indeed when compared with chromatographic analytical methods such as HPLC and/or GC/MS^31,33^. Our results indicate that both approaches have merit and indeed allow analysis of different aspects of sample chemistry.

### GC/MS analysis of plant ethanol soluble metabolites

Primary metabolites (e.g. amino acids, sugars and organic acids) and secondary metabolites (e.g. phenols, flavonoids and sterols) are components of the complex networks of biochemical pathways in plant metabolism^34^. Profiling their relative concentrations in samples provides information directly related to plant biological behaviour in response to changes in physiological or environmental conditions e.g. availability of important mineral nutrients such as nitrogen^35,36^ and phosphorus^37^, or to differences in composition between tissues e.g. the shoot and root terpenoid profiles of *Tanacetum vulgare*^38^.

### Analysis of sample total ion chromatographic data

Visual analysis of the mean total ion chromatograms (TIC) of MOX-TMS derivatised ethanolic extracts of the leaf and stem samples (n = 54) showed many of the major metabolite peaks were common to samples from both tissues, and substantive differences between them were not apparent (Fig. S4A,B). PCA of these data, using a pre-processing regime of Log10 transformation (to scale for the great differences observed in peak height), automatic Whittaker filter baseline correction, normalisation to unit area (1-norm), and mean centring; and cross validation by venetian blinds protocol (7 data splits), revealed difference between sample type to be a major source of variance along PC axis 2, and the loadings for this model were highly complex (Fig. S4C,D).

**Figure 3:**
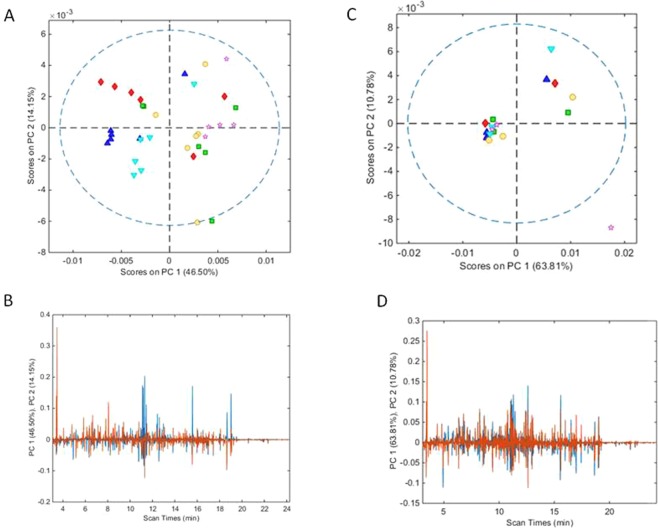
(**A**,**B**) Show the scores and loadings plots for PCA models based on TICs of MOX-TMS derivatised ethanolic extracts of ground rosemary leaf samples after analysis by GCMS whilst (**C**,**D**) show the scores and loadings plots for a PCA model based on the stem data (**A**,**C** - red diamonds = CH; green square = CL; dark blue triangle PH; light blue inverted triangle = PL; pink star = control; yellow circle = InOrg; dotted line = 95% confidence level; (**C**,**D**) - loading plot for PC1 = blue line and PC2 = brown line). CH: compost from anaerobic digested cattle slurry at dose 60 t/ha; CL: compost from anaerobic digested cattle slurry at dose 30 t/ha; PH: compost from anaerobic digested pig slurry at dose 60t/ha; PL: compost derived anaerobic digested pig slurry at dose 30t/ha; InOrg: inorganic fertiliser.

PCA was repeated on the mean TIC data after separation into stem and leaf datasets using protocols for cross-validation and pre-processing identical to the PCA of the entire TIC data set. PCA of the leaf TICs resulted in a 6-component model that explained 83.88% of total variance (RMSEC and RMSECV of 3.96 × 10^−5^ and 5.65 × 10^−5^, respectively). Plotting the scores for PC1 (46.5% variance) vs PC2 (14.15% variance) showed partial grouping with the rosemary plant samples from the treatment with composted pig slurry digestate samples (PH and PL), generally having negative scores in contrast to samples from the control and InOrg treatments, which generally had positive scores on PC1 (Fig. 3A). The samples treated with composted cattle manure separated along PC axis 1 with the high dose group generally having negative scores, and the low dose group positive scores along PC1. These results showed treatment with pig slurry digestate compost had a consistently major effect on leaf composition, compared to the control treatments, and also that there was an effect of the dose in the treatment with composted cattle manure digestate, with the low dose samples grouping with the control and the inorganic treatments.

However, PCA of the stems sample data showed less evidence of treatment effect. A 5-component PCA model explained 90.39% of total variance (RMSEC and RMSECV of 4.29 × 10^−5^ and 8.81 × 10^−5^ respectively). The scores plot for PC1 (63.81% variance) vs PC2 (10.78% variance) failed to separate the samples into groups according to treatment (Fig. 3C) and both axes largely explained within group variation. Attempts to improve stem sample score grouping by sample removal or alternative data pre-treatment steps e.g. external parameter orthogonalisation (EPO) to diminish within group variance, failed to improve clustering of the stem samples by experimental treatment (data not shown). Loadings plots for PC1 for both stem and leaf TIC data are shown in Fig. 3B,D, and are highly complex.

### Analysis of deconvoluted metabolite abundances in the experimental samples

Given the complexity of the TIC data and loading resulting from PCA it was decided to re-analyse the data at the level of individual chemical components to investigate if by this approach it would be possible to detect changes in sample chemistry that correlated with fertiliser treatment. The GC/MS TIC data were deconvoluted using AMDIS (Automated Mass Spectrometry Deconvolution and Identification System, NIST). This process amounts to an algorithmic separation of co-eluting components by identification of co-eluting masses and allows a clean, if not pure mass spectrum to be obtained for components in a complex mixture^39^. Deconvolution greatly facilitates the accuracy of subsequent mass spectral library matching, and has the additional benefit of reducing data dimensionality by converting the many data points of the original TIC data into chemically more meaningful variables^39^. A rosemary mass spectral target compound library was constructed in AMDIS that represented the chemistry of the leaf and stem samples. The library was obtained by deconvolution and subsequent library matching of GC/MS data from 3 randomly selected leaf and 3 stem samples, using settings of low sensitivity, and medium resolution and peak shape requirement^40^. The resulting library contained spectra of 160 deconvoluted chromatographic peaks, which were identified tentatively by library match and visual comparison using MS Search 2.0 (Table S1). The majority of the peaks identified in the TICs were either known soluble plant metabolites or unknown mass tags that had been reported in the literature previously. Some peaks were recognised as derivitisation artefacts; these were not excluded from the library as it was important to test for differences in derivitisation efficiency between the sample groups.

Batch processing of the chromatographic/mass spectral data for the stem and leaf samples (n = 54) in AMDIS using the rosemary target library resulted in a compound table for each sample, with the concentration of each library metabolite detected in the sample being expressed as a percentage of the normalised total ion current. The compounds identified in the sample TICs represented more than half of the ion current (mean area = 51.68%, standard deviation (SD) = 5.37%) with the remaining current being a mix of baseline noise and metabolites present in the samples at very low concentrations. The individual compounds ranged in mean abundance from 7.08% of total ion current (a methoxytrimethylsilanamine derivitisation artefact with a retention time (RT) of 3.46 min) to 0.007% for trace amounts of a compound tentatively identified as sulphuric acid at 5.39 min.

PCA of these data following log_10_ transformation and mean centring resulted in a 2 component model that explained 44.65% of data variance (RMSEC and RMSECV of 0.3624 and 0.4060 respectively). PCA cross-validation was made using a venetian blinds protocol with 10 data splits. A bar chart of the metabolite abundancy data used for modelling is shown in Fig. S5A, and there are obvious similarities with the mean TIC data plot shown in Fig. S4A,B as the compounds in the rosemary library correspond with the main peaks present in the TIC data. Scores on PC1 (26.17% variance) clearly resolved the leaf and stem samples (Fig. S5B) but some grouping according to experimental treatment was evident along PC axis 2, which explained 18.49% of variance (Fig. S5C). Removing variables corresponding to likely derivitisation artefacts or to unknown mass tags failed to improve model fit (RMSEC and RMMSECV of 0.3716 and 0.4169 respectively) or grouping according to experimental treatment (data not shown). Analysis of the loading for PC1 (Fig. S5D) suggested that metabolites tentatively identified as ribose, glucose, salicylic acid glucoside, glutaric acid and galactinol (metabolite IDs of 10, 77, 27, 139 and 23 respectively in Table S1) were greater in the leaf samples whilst levels of sucrose, coniferyl alcohol, 4-trans-caffeoylquinic acid, trimethyl-silanol phosphate, and an unknown mass tag MST 3814.8 correlated with the stem samples (metabolite IDs of 37, 59, 67, 157 and 111 respectively in Table S1).

**Figure 4:**
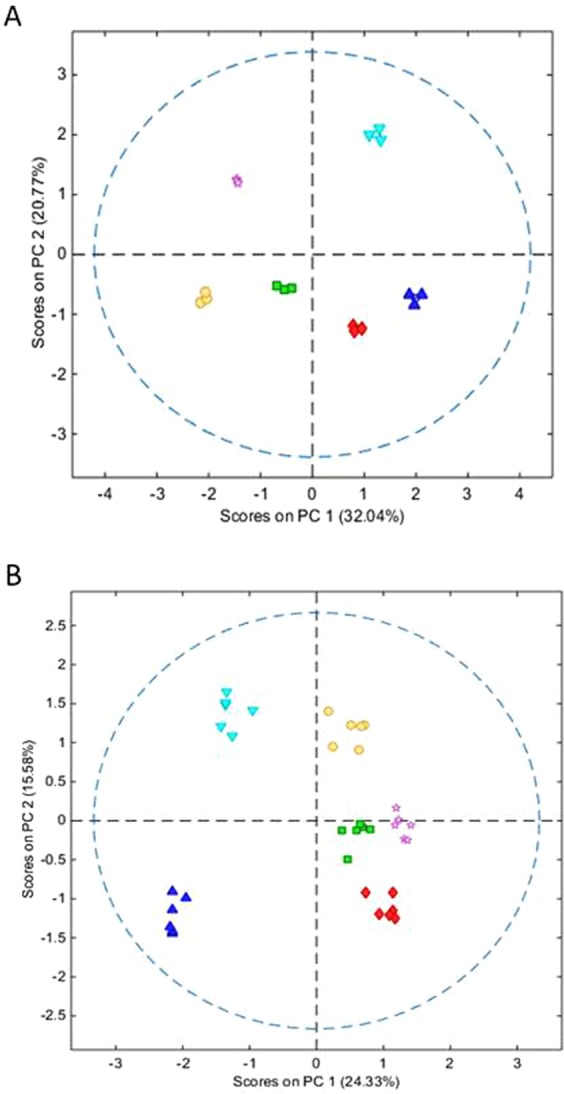
(**A**,**B**) Show the scores plot for PCA models based on extracted metabolite abundances in the stem samples respectively. Data were log 10 transformed, GLS weighted according to experimental class and mean centred (red diamonds = CH; green square = CL; dark blue triangle PH; light blue inverted triangle = PL; pink star = control; yellow circle = InOrg; dotted line = 95% confidence level). CH: compost from anaerobic digested cattle slurry at dose 60 t/ha; CL: compost from anaerobic digested cattle slurry at dose 30 t/ha; PH: compost from anaerobic digested pig slurry at dose 60t/ha; PL: compost derived anaerobic digested pig slurry at dose 30t/ha; InOrg: inorganic fertiliser.

PCA modelling of the leaf and stem metabolite data sets independently with a pre-processing protocol involving log_10_ transformation, general least squares (GLS) weighting (weighted to treatment class, alpha = 1.00) to help reduce within class variance, followed by mean centring allowed better sample grouping to experimental treatment. PCA of the stem sample data resulted in a 2-component model that explained 52.81% of the variance in the data (RMSEC and RMSECV of 0.1412 and 0.3690 respectively). A scores plot of PC1 vs PC2 resolved completely the samples according to experimental treatment (Fig. 4A). The relatively low amount of variance explained by PC1 is due to the GLS filtering algorithm by which variance associated with within group variance is excluded from the model. The stem samples treated with composted pig slurry digestate (PH and PL) and samples treated with the high rate of composted cattle manure digestate (CH) had positive scores along PC axis 1 (32.04% variance), correlating with high levels of compounds tentatively identified as quinic acid, D-glucopyranoside or D fructofuranosyl, sorbitol and two unknown mass tags (metabolite IDs 112, 1, 58, 110 and 92 respectively in Table S1). In contrast, the stem samples corresponding to the low dose of cattle manure digestate compost (CL) and more so, samples from the control and inorganic treatments had negative scores on this axis, correlating with levels of sucrose, salicylic acid, xylose, threonic acid and an unknown mass tag (metabolites IDs of 37, 135, 70, 36 and 101 respectively in Table S1). Furthermore, the low dose of composted pig slurry digestate (PL) treated stem samples and the control stem samples had positive scores along PC axis 2 (20.77% variance), correlating with higher concentrations of metabolites tentatively identified as sucrose, D-glucopyranoside or D-fructofuranosyl, quinic acid, maltose and sorbitol (metabolite IDs of 37, 1, 112, 73 and 58 respectively in Table S1), whilst the other stem sample groups had negative scores in PC2 correlating with greater concentrations of galactinol, sucrose, glucose, anhydroglucose and an unknown mass tag (metabolite IDs of 23, 120, 118, 48 and 104 respectively in Table S1).

PCA of the leaf sample metabolite data using identical pre-processing and cross validation protocols resulted in a 2-component model explaining 39.91% variance (RMSEC and RMSECV of 0.1573 and 0.2765 respectively). Leaf samples treated with composted pig slurry digestate at both high and low dosage had negative scores in PC1 (24.33% variance) correlating with high levels of metabolites tentatively identified as glucose, glycerol, cellobiose, maltose, malic acid and 2-oxoglutaric acid (metabolite IDs of 77, 160, 148, 73 and 30 respectively in Table S1). The remaining leaf sample groups had positive scores along this axis correlating with metabolites tentatively identified as saccharic acid, sucrose, maltose, O-Glycerol-beta-D-galactopyranoside and 1-trans-caffeoylquinic acid (metabolite IDs of 132, 37, 121, 33 and 68 respectively in Table S1). The scores of the leaf samples along PC2 (15.58% variance) also correlated with treatment group. The scores of the leaf samples from the high dose of both compost had negative values on component axis 2 compared with leaf samples corresponding to treatment with low dose of composted cattle manure digestate or the control, which were relatively neutral in PC2, inferring greater amounts of xylobiose, hydrocaffeic acid, galactose, 1-methyl galactopyranoside and an unknown mass tag (metabolite IDs of 147, 145, 21, 40 and 80 respectively in Table S1). In contrast, the leaf sample groups from the low dose pig slurry compost (PL) and with the inorganic (InOrg) treatment had positive scores in PC2 correlating with relatively greater concentrations of 2-oxoglutaric acid, saccharic acid, maltose, glucose and 2-O-Glycerol-beta-D-galactopyranoside (metabolite IDs of 139, 132, 121, 77 and 33 respectively in Table S1).

In summary, the concentrations of sucrose, glucose and maltose and other metabolites in the rosemary samples displayed highly complex relationships to fertiliser treatment, with no clear pattern being discernable. A similar observation was reported by Fernie and Urbanczyk-Wochniak^36^, in a study of tobacco plants under different nitrogen regimes. In this case, levels of fructose, galactose, raffinose and sucrose were not clearly related to nitrate nutrition. Furthermore, the changes in carbohydrate abundance depending on the N availability (saturation or deficiency) seemed to be tissue-specific and dependent on the carbohydrate species, since the behaviour observed was different for each carbohydrate. This fact was also reported in the leaves and roots of tomato under plants grown under nitrogen, phosphorus or potassium-deficient condition^41^ and in leaves from pak choi plants grown in a soil amended with composts of different origin^42^. The concentration of other types of compounds, phenolic compounds (eg caffeoylquinic acid, galactopyranoside, hydrocaffeic acid) also presented different responses to the different soil treatments and that might be partly due to the various nitrogen contents of these composted digestates. At this regard, Neugart *et al*. (2018) have recently concluded that nitrogen might not be the only factor influencing the leaf concentration of monoacylated kaempferol glycoside in plants grown on soils amended with three different composts. Several studies, for example, have reported 2-oxoglutaric acid (synonyms α-ketoglutarate, 2-ketoglutaric acid, or 2-oxoglutarate) to have an important role as a key regulator at the intersection between the carbon and nitrogen metabolic pathways^43,44^. Fernie and Urbanczyk-Wochniak (2004)^36^ observed large decreases in 2-oxoglutaric acid in tobacco plants grown on limited nitrogen supply. On the other hand, Sung *et al*. (2015) related the low levels of 2-oxoglutaric acid found in leaves of tomato plants to P deficiency. In our experiment, higher levels of this metabolite appeared associated to the low dose of composted pig slurry digestate and the inorganic treatment, which can be ascribable to the higher availability of nitrogen and phosphorus in these treatments, especially in the inorganic treatment. 2-oxoglutaric acid and glutamate are key regulators of amino acid biosynthesis^45^, and our observation of increased concentrations of 2-oxoglutaric acid on high-nitrogen treatments is in agreement with other studies that reported this component to be increased, together with a wide group of amino acids, under conditions of nitrogen saturation^36^.

### TGA proximate analysis

Plant biomass contains large amounts of the cell wall components hemicellulose, cellulose, pectin, and lignin, together with primary and secondary metabolites proteins storage polymers such as starch and lipid. Analysis of plant material by thermogravimetry means provides information on the thermal stability of the various chemical components in the samples and in this study, insight into changes in composition effected by different fertilizer treatments. The calculation of mass fraction in the samples due to moisture, volatiles, fixed carbon and ash is called proximate analysis. Sample pyrolysis generally takes place in a non-oxidising atmosphere to avoid the occurrence of combustion, and the process can be divided into three stages with regards to weight loss and temperature. The first stage occurs between 30 to 120 °C with maximal mass loss occurring at about 110 °C. During this stage, moisture evaporates from the samples. The second stage occurs between approximately 200 °C and 600 °C, and is attributable to loss of labile or volatile components of the biomass. The cell wall components hemicellulose, pectin and cellulose thermally decompose at these temperatures together with other labile metabolites and polymers. Cellulose has greater thermal stability and woody samples often show biphasic mass loss during this stage of thermal decomposition. The mass remaining after the loss of volatiles is classified as char, and mostly this is comprised of condensed lignin together with the inorganic fraction of the biomass. The amount of fixed carbon in the samples can be calculated by subtraction of the weight of ash from the weight of char^46^.

**Figure 5:**
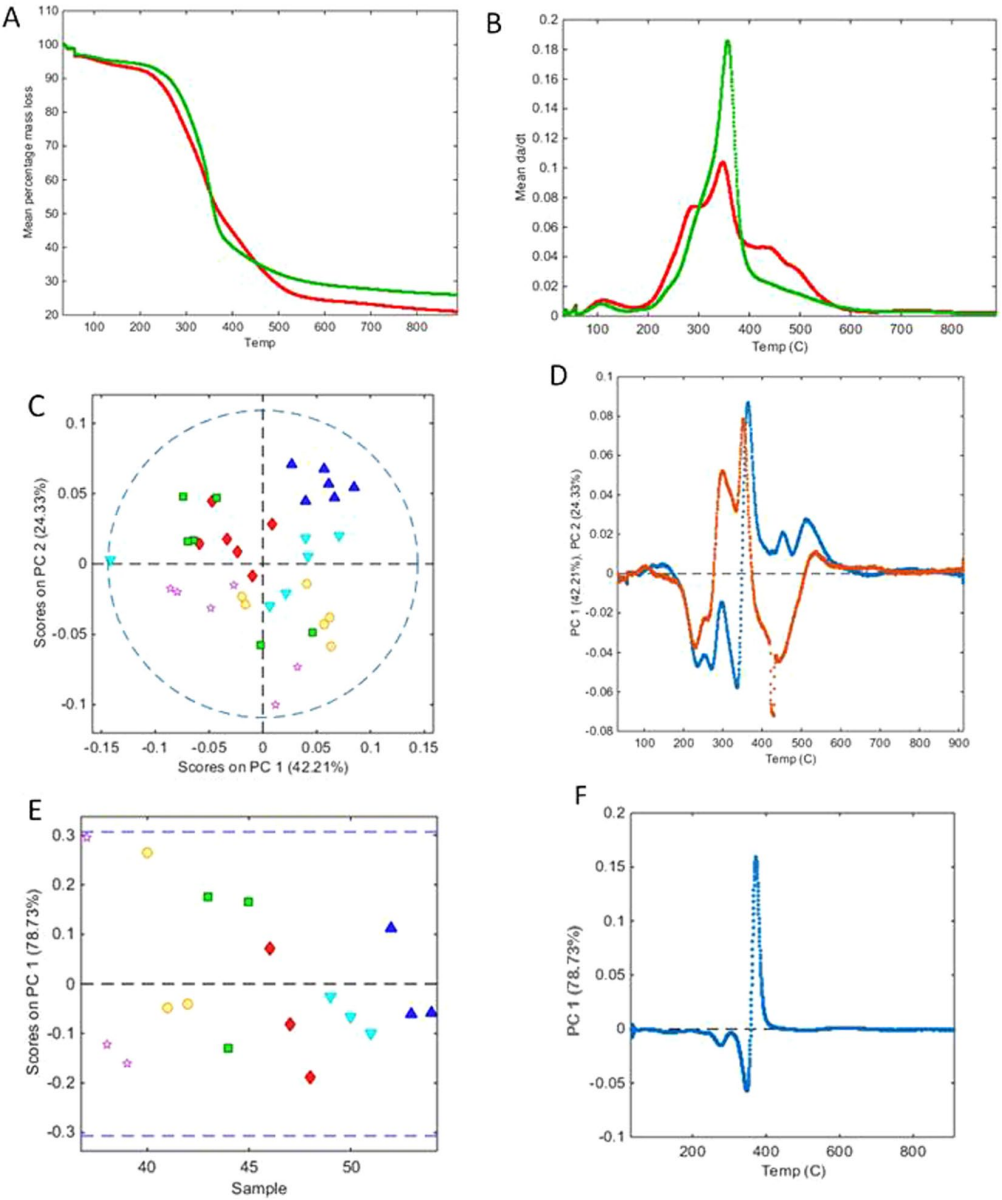
Mean percentage mass loss (**A**) or derivative mass loss (**B**) plotted against temperature during thermogravimetric analysis of leaf (red) and stem samples (green) respectively. (**C**) PCA scores plot of leaf TGA data (red diamonds = CH; green square = CL; dark blue triangle PH; light blue inverted triangle = PL; pink star = control; yellow circle = InOrg; dotted line = 95% confidence level). (**D**) Leaf sample PCA loadings for PC1 (blue) and PC2 (brown). (**E**) PCA scores plot of stem TGA data (red diamonds = CH; green square = CL; dark blue triangle PH; light blue inverted triangle = PL; pink star = control; yellow circle = InOrg; dotted line = 95% confidence level). (**F**) Stem sample PCA loadings for PC1 (blue) and PC2 (brown). CH: compost from anaerobic digested cattle slurry at dose 60 t/ha; CL: compost from anaerobic digested cattle slurry at dose 30 t/ha; PH: compost from anaerobic digested pig slurry at dose 60t/ha; PL: compost derived anaerobic digested pig slurry at dose 30t/ha; InOrg: inorganic fertiliser.

Proximate analysis of the samples revealed differences in thermal decomposition between the two tissues in the region corresponding to loss of volatiles. The rate of mass loss for the leaf samples was less than that observed for the stem samples, although ultimately more mass was lost from the leaf samples during analysis, indicating greater volatile content in the leaf samples (Fig. 5A). Differential plots of these data (DTG curves) showed the leaf samples had a more complex thermal decomposition compared to the stem samples (Fig. 5B), probably due to the presence of a greater amounts of thermally labile metabolites and storage polymers. Whilst the leaf and stem samples displayed maximal mass loss (Tmax) at very similar temperatures (355 °C and 346 °C for stem and leaf samples respectively), the leaf samples showed additional mass loss events at 289 °C and 435 °C that were less evident in derivative plots of the stem samples. These differences imply a direct impact on thermal decomposition characteristics by the specific distribution of organic and inorganic constituents among plant parts and agree with the findings of Damartzis *et al*.^24^.

The mass loss event due to loss of moisture from the samples detected at approximately 110 °C was very similar for samples of both tissue types indicating both types of sample had dried comparably. After correcting the mass loss data for moisture content (DM), analysis of variance (Anova) detected significant differences between leaf and stem sample means for volatile content (DM Vol; means of 79.68% and 74.16% for leaf and stem samples respectively; P < 0.001; s.e.d. 0.227); content of fixed carbon (DM FC, means of 13.36% and 20.55% for leaf and stem samples respectively; P < 0.001; s.e.d. 0.145); ash content (DM Ash, means of 6.96% and 5.28% for leaf and stem samples respectively; P < 0.001; s.e.d. 0.184); and Tmax (means of 346 °C and 355 °C for leaf and stem samples respectively; P < 0.001; s.e.d. 11.80). Significant differences were also detected by ANOVA between means of volatile and fixed carbon content of leaf and stem samples after correcting the data for ash content (DMAF Vol; means of 85.64% and 78.30% for leaf and stem samples respectively; P < 0.001; s.e.d. 0.183) and DMAF FC (means of 14.36% and 21.70% for leaf and stem samples respectively; P < 0.001; s.e.d. 0.183).

The higher content of fixed carbon in stem samples maybe be due to higher lignin, and cellulose content, corresponding to greater vascularization, as it has been reported lignin promotes the production of fixed carbon during pyrolysis^47^. The lower ash content in the stem samples might also influence sample fixed carbon content as ash has a strong catalytic effect to pyrolysis and hampers the generation of fixed carbon^48^.

Anova of TGA data from the leaf samples only (Table 2) detected significant differences between treatment means for moisture, ash, volatiles and fixed carbon, and Tmax. Ash content in the samples, which correlates with relates the proportion of inorganic minerals in the samples^49^, was increased significantly by application of inorganic fertiliser or manure relative to the control samples, with pig manure at high dose having the greatest effect. Treatment with inorganic fertiliser or manure generally reduced fixed carbon content significantly. On the other hand, the volatile fraction generally increased after application of fertilizer but it is difficult to conclude from these data whether there are substantial differences in volatile content as a result of fertilizer treatment.

**Table 2:** Anova predicted means of leaf sample TGA data together with values of P and standard error. Means are given as percentage composition (AR = as received, DM = dry matter corrected, or DMAF = dry matter ash free corrected) or by mean temperature for thermal decomposition events, ns denotes not significant. CH: compost from anaerobic digested cattle slurry at dose 60 t/ha; CL: compost from anaerobic digested cattle slurry at dose 30 t/ha; PH: compost from anaerobic digested pig slurry at dose 60t/ha; PL: compost derived anaerobic digested pig slurry at dose 30t/ha; InOrg: inorganic fertilisation.

Leaf Samples	PL	PH	CL	CH	InOrg	Control	P	s.e.d
AR Ash	6.84%^b,c^	7.50%^c^	6.61%^b^	7.03%^b,c^	6.9%^b,c^	5.48%^a^	<0.001	0.309
AR Fixed carbon	12.22%^a^	12.31%^a,b^	13.19%^c,d^	12.92%^a,b,c^	13.00%^b,c^	13.85%^d^	<0.001	0.341
AR Volatile	77.86%^b^	77.10%^a,b^	76.72%^a^	76.61%^a^	76.55%^a^	77.20%^a,b^	0.015	0.378
DM Ash	7.05%^b,c^	7.74%^c^	6.85%^b^	7.28%^b,c^	7.18%^b,c^	5.68%^a^	<0.001	0.319
DM Fixed carbon	12.61%^a^	12.70%^a,b^	13.66%^c,d^	13.38%^b,c^	13.47%^c^	14.35%^d^	<0.001	0.344
DM Volatile	80.34%	79.57%	79.49%	79.34%	79.35	79.97%	ns	0.427
DMAF Fixed carbon	13.56%^a^	13.76%^a,b^	14.66%^c^	14.43%^b,c^	14.51%^b,c^	15.21%^c^	0.001	0.364
DMAF Volatile	86.44%^c^	86.24%^b,c^	85.34%^a^	85.57%^a,b^	85.49%^a,b^	84.79%^a^	0.001	0.364
Tmax (289 °C)	291.1 °C^b^	291.0 °C^b^	287.2 °C^a^	287.3 °C^a^	289.9 °C^a,b^	287.8 °C^a^	0.011	1.364
Tmax (346 °C)	346.7 °C^c^	346.5 °C^b,c^	344.4 °C^a,b^	344.2 °C^a^	347.4 °C^c^	344.6 °C^a,b^	0.007	0.985
Tmax (435 °C)	436.7 °C^c^	432.5 °C^a^	435.6 °C^b,c^	433.6 °C^a,b^	436.6 °C^c^	436.7 °C^c^	0.001	1.107

Anova detected that the temperature of peak mass loss at approximately 346 °C, which most likely corresponds to thermal degradation of cellulose^50^, increased with application of inorganic fertiliser (+2.8 °C). A smaller increase in Tmax (~2 °C) was detected as significant for the pig manure treated samples whilst the effect of cow manure application on Tmax was not detected as significant compared to the control treatment. A similar increase in temperature was detected for the minor thermal decomposition event at approximately 289 °C, which most likely corresponds to hemicellulose decomposition^50,51^. Treatment with inorganic fertiliser increased the peak temperature of this event (Tmax 289 °C) by nearly 2 °C. Furthermore, samples from pig manure treated plants were increased by approximately 4 °C higher compared with control. In contrast, the samples treated with cattle manure were not detected as being different from the control samples.

The observed differences in maximum temperature of mass loss can be associated to differences in the composition between leaves from plants grown in the six treated soils. Increased Tmax at 289 °C and 346 °C may be due to lower hemicellulose and cellulose content, which caused them to react more slowly^52^. Another explanation could be that the hemicellulose and cellulose in these samples displayed greater thermal stability^52,53^ perhaps due to changes in hemicellulose branching, cellulose packing or crystallinity, or the presence of increased amounts of storage or structural proteins.

Anova of the stem sample data detected no significant changes between treatment groups in the proportion of ash, volatiles or fixed carbon in the samples, or any significant shifts in the temperature of peak mass loss, which occurred at approximately 355 °C (data not shown).

**Figure 6:**
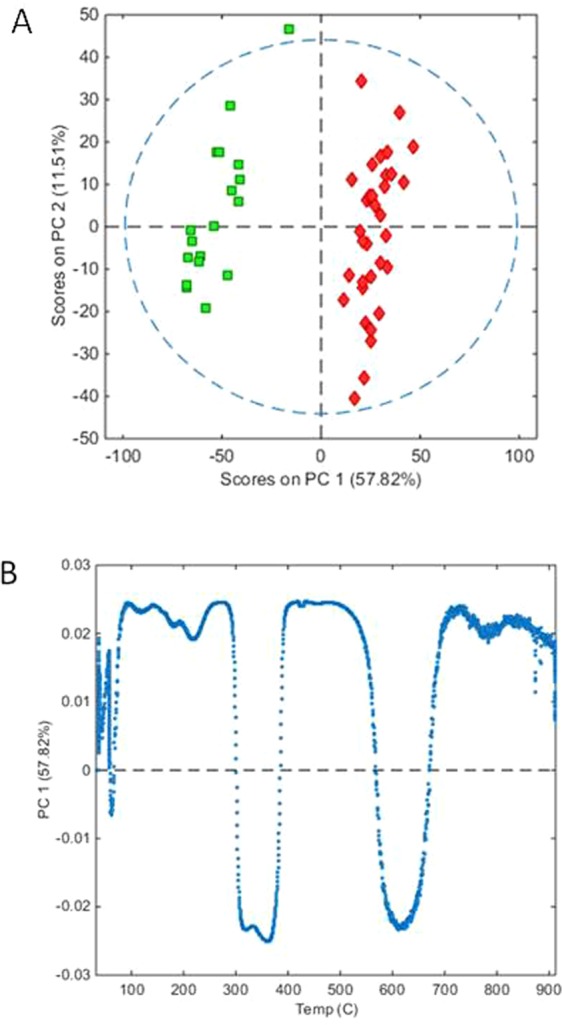
(**A**) PCA scores plot of leaf and stem TGA data (leaf = red diamonds, stem = green squares), whilst (**B**) shows the loadings for PC1.

PCA of the leaf and stem TGA data with venetian blinds cross validation and data autoscaling gave a 3 component model that explained 95.91% of variance (RMSEC and RMSECV of 0.2003 and 0.5176 respectively) and allowed complete separation of the leaf and stem sample scores along PC 1 and PC2 component axes (76.57% and 14.07% variance respectively). The separation of the two tissue types by this model (not shown) was associated with scores on PC axis 1 correlating to the region extending between 354 °C and 445 °C where the two tissues displayed differential mass loss. Modelling of the data after differentiation (*da/dt*) also indicated that the temperatures associated with peak thermal decomposition were important discriminators between leaf and stem samples. PCA gave a 4 component model that explained 83.43% of variance (RMSEC and RMSECV of 0.4015 and 0.7578 respectively) and a scores plot for PC 1 and PC2 component axes (57.82% and 11.51% variance respectively) allowed complete separation of the leaf and stem sample scores (Fig. 6A). In this case, leaf samples were associated with positive scores and stem with negative scores along PC axis 1, which displayed discriminatory negative loadings at 2 broad peaks. The first of these was a doublet at 302–384 °C, corresponding with the thermal transitions detected at 289 °C and 346 °C, and in addition the stem samples were associated with a second broad peak between 573–664 °C which had not been detected as discriminatory in the model of the undifferentiated TGA data (Fig. 6B).

PCA of just the leaf sample TGA data after autoscaling and using venetian blinds cross validation resulted in 4 component model that explained 86.39% of variance (RMSEC and RMSECV of 0.006 and 0.001 respectively) and a scores plot of PC1 against PC2 (42.21% and 24.33% of variance respectively) showed that, with one exception, the samples from both pig manure treatments have in higher scores along principal component axis 1 compared with those from the cattle manure treatments. The InOrg samples also had to the main higher scores along this axis compared with the control samples (Fig. 5C). These positive scores correlated with differences primarily at 364 °C and to a lesser effect at 454 °C and 512 °C, whilst negative scores along this axis corresponded with differences at 236 °C, 271 °C and 336 °C (Fig. 5D). Principal component axis 2 largely served to separate InOrg and control from manure treatment samples, with positive scores being associated with variance at 298 °C and 353 °C, and negative scores, which were predominantly shown by both control groups, with variance at 227 °C, 429 °C and 445 °C (Fig. 5D). Modelling just the stem TGA data by PCA using identical pre-processing and cross validation protocols resulted in a 1 component model that explained 78.73% of variance (RMSEC and RMSECV of 0.001 and 0.002 respectively). Analysis of the sample scores for the stem samples showed very little grouping by experimental treatment (Fig. 5E) with the difference in variance occurring at 372 °C (positive) and 347 °C (negative), see Fig. 5F. These results suggest changes in plant thermal stability, which may have implications for crop processing are dependent on cultivation practice, and which need further investigation.

## Conclusions

The results obtained with FTIR and GC/MS revealed a clear differentiation between organic and inorganic fertilising treatments on the metabolite content of rosemary plants (*Rosmarinus officinalis* L.), and these differences are tissue dependant. GC/MS has shown that the leaves of the rosemary plants of the treatment with the compost derived from pig slurry digestate (P) differed in metabolite composition compared to the rest of treatments. In addition, a clear effect of the dose in the treatment with composted cattle manure digestate was observed, with the low dose being more similar in effect to the control and the inorganic treatments. Changes in metabolite composition are most likely brought about by N availability, and whilst the differences detected were tissue and metabolite specific, no clear trends relating concentration and fertiliser regime were apparent. Higher concentrations of 2-oxoglutaric acid were associated with the low dose of composted pig slurry digestate and the inorganic treatment, probably due to the higher availability of nitrogen in these treatments, especially in the inorganic treatment. Proximate analysis of the leaf and stem samples revealed differences between the two tissues in the thermal decomposition of the volatile fraction. Moreover, TGA data from the leaf showed that fixed carbon was decreased in samples treated with either inorganic fertiliser or composted pig slurry digestate). Lastly, differences in volatile fraction thermal stability were detected between leaf samples treated with either inorganic or composted pig slurry digestate compared with control and composted cattle manure digestate treated leaves.

Thus, this study provides new insight into metabolic and chemical changes in rosemary plants responding to different fertilising treatments. Furthermore, it demonstrates the value of taking a multidisciplinary approach when studying plant response to changes in growing conditions.

## Supplementary information

Supplemantary information
