# Supplementary material for: The effect of anaerobic digestate derived composts on the metabolite composition and thermal behaviour of rosemary
Source: Sci Rep. 2019 Apr 24;9:6489. doi: 10.1038/s41598-019-42725-6 (PMC6482180; doi:10.1038/s41598-019-42725-6)
Supplement: Supplementary file 1 — Supplemantary information [file 41598_2019_42725_MOESM1_ESM.docx]

**The effect of anaerobic digestate derived composts on the metabolite composition and thermal behaviour of rosemary**

Bustamante, M.A., Nogués, I., Jones, S., Allison, G.G.

# Supplementary materials

# Figure S1

PCA of mid IR ATR spectra from ground leaf and stem samples: A. Spectra of samples. B. Scores plot (red diamonds = leaf samples; green squares = stem samples; dotted line = 95% confidence level). C. Loading plot for PC1. CH: compost from anaerobic digested cattle slurry at dose 60 t/ha; CL: compost from anaerobic digested cattle slurry at dose 30 t/ha; PH: compost from anaerobic digested pig slurry at dose 60t/ha; PL: compost derived anaerobic digested pig slurry at dose 30t/ha; InOrg: inorganic fertilisation.

# Figure S2

PCA of mid IR transmission spectra from ethanolic extracts of leaf samples dried onto silicon plates: A. Spectra of samples. B. Scores plot for PC1 vs. PC2 (red diamonds = CH; green square = CL; dark blue triangle PH; light blue inverted triangle = PL; pink star = control; yellow circle = InOrg; dotted line = 95% confidence level). C. Loading plot for PC1. CH: compost from anaerobic digested cattle slurry at dose 60 t/ha; CL: compost from anaerobic digested cattle slurry at dose 30 t/ha; PH: compost from anaerobic digested pig slurry at dose 60t/ha; PL: compost derived anaerobic digested pig slurry at dose 30t/ha; InOrg: inorganic fertilisation.

# Figure S3

PCA of mid IR transmission spectra from ethanolic extracts of stem samples dried onto silicon plates: A. Spectra of samples. B. Scores plot for PC1 vs. PC2 (red diamonds = CH; green square = CL; dark blue triangle PH; light blue inverted triangle = PL; pink star = control; yellow circle = InOrg; dotted line = 95% confidence level). C. Loading plot for PC1. CH: compost from anaerobic digested cattle slurry at dose 60 t/ha; CL: compost from anaerobic digested cattle slurry at dose 30 t/ha; PH: compost from anaerobic digested pig slurry at dose 60t/ha; PL: compost derived anaerobic digested pig slurry at dose 30t/ha; InOrg: inorganic fertilisation.

# Figure S4

Mean TIC of leaf (A) and stem (B) following analysis by GCMS. C shows the scores plot for the PCA model based on TIC data from leaf (red diamonds) and stem (green squares) data. D shows the loadings for PC1 (blue line) and PC2 (orange line).

# Figure S5

A: Bar chart showing the mean abundance of the metabolite variables extracted from the ms data. B and C: Scores plots of PC1 (26.17% variance) plotted against PC2 (18.49% variance) for PCA of metabolite extracted abundances in the leaf and stem samples coloured by (B) tissue (leaf =red diamonds, stem =green squares), or by (C) treatment (red diamonds = CH; green square = CL; dark blue triangle PH; light blue inverted triangle = PL; pink star = control; yellow circle = InOrg; dotted line = 95% confidence level).D shows the loadings for the metabolite variables vs retention time. CH: compost from anaerobic digested cattle slurry at dose 60 t/ha; CL: compost from anaerobic digested cattle slurry at dose 30 t/ha; PH: compost from anaerobic digested pig slurry at dose 60t/ha; PL: compost derived anaerobic digested pig slurry at dose 30t/ha; InOrg: inorganic fertilisation.

# Figure S1

# Figure S2

# Figure S3


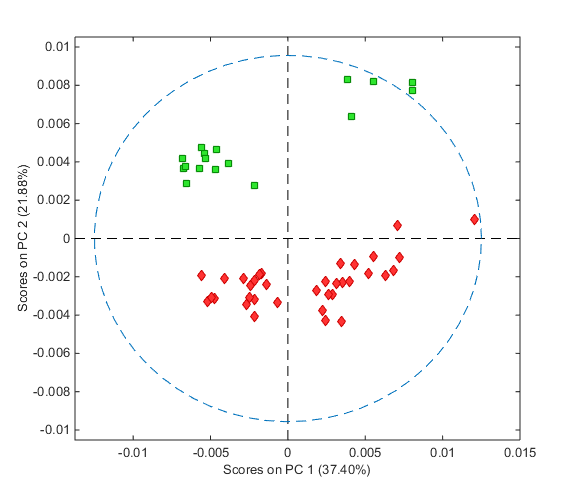

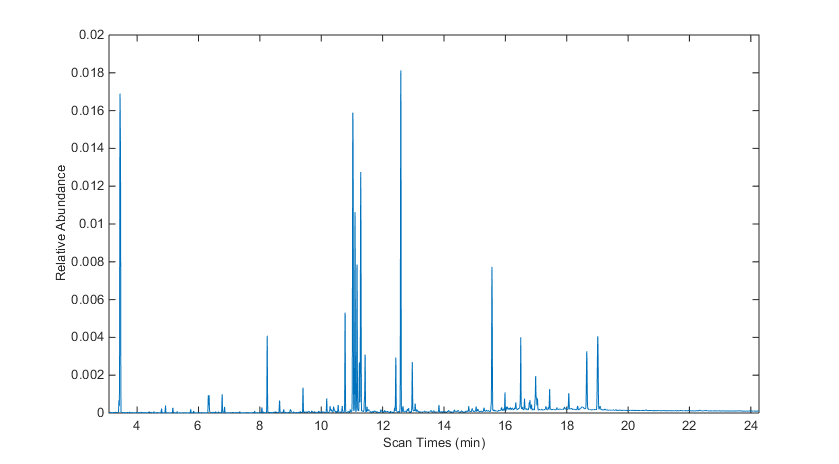


B

A

C

D


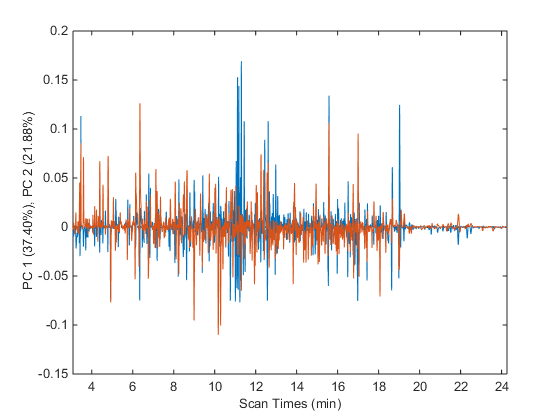

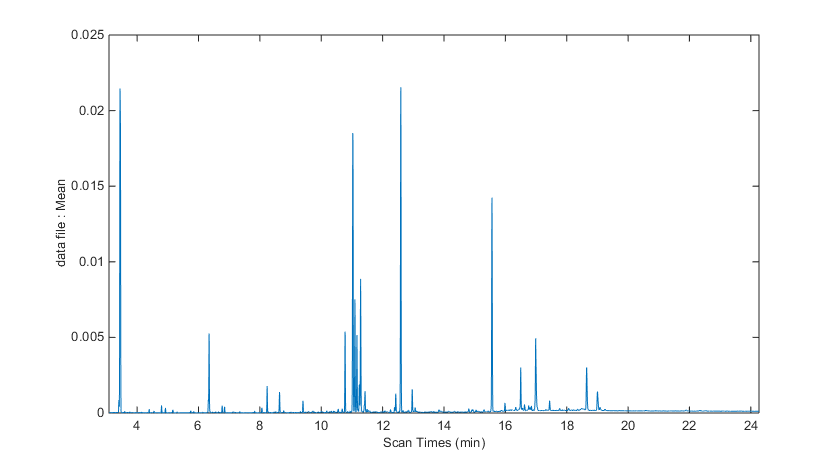


# Figure S4

# Figure S5

|  | Table S1 Annotated peaks, their retention time and mean relative amount in the GC/MS TICs from the stem and leaf samples after mass spectral deconvolution and library identification. Compounds are shown in order of mean percentage relative abundance. |  |  |
| --- | --- | --- | --- |
| **ID** | **Metabolite name** | **RT (min)** | **Mean Relative Amount** |
| 155 | Silanamine, N-methoxy-1,1,1-trimethyl-N-(trimethylsilyl)- | 3.4576 | 7.0826% |
| 126 | M000060_A209002-101_METB_0_PHYTOH_Inositol, myo- (6TMS) | 12.606 | 6.3113% |
| 113 | M000007_A185001-101_METB_577900_EINS_Quinic acid, D(-)- (5TMS) | 11.0294 | 5.5202% |
| 77 | glucose (penta TMS/MOX derivitive) | 11.2849 | 3.3920% |
| 142 | M000606_A187002-101_METB_1869.2_EISTR_Fructose, D- (1MEOX) (5TMS) | 11.0993 | 3.1488% |
| 37 | EIQTMS_N12C_LJAN_2654.8_2233BN14_Sucrose (8TMS); alpha-D-Glc-(1,2)-beta-D-Fru | 15.5718 | 2.5602% |
| 14 | EIQTMS_N12C_ATHL_1883.9_3161BN26_Fructose methoxyamine {BP} (5TMS) | 11.1636 | 2.3998% |
| 39 | EIQTMS_N12C_LJARL_1828.7_2236BN14_Citric acid (4TMS) | 10.7785 | 1.8213% |
| 17 | EIQTMS_N12C_ATHL_3392.6_3161BN25_Raffinose (11TMS); alpha-D-Gal-(1,6)-alpha-D-Glc-(1,2)-beta-D-Fru | 18.6499 | 1.5427% |
| 1 | ?-D-Glucopyranoside, 1,3,4,6-tetrakis-O-(trimethylsilyl)-?-D-fructofuranosyl 2,3,4,6-tetrakis-O-(trimethylsilyl)- | 15.5597 | 1.4785% |
| 46 | EIQTMS_N12C_NID1_3098.7_1267BK12_[822; Caffeic acid (3TMS)] | 19.0038 | 1.3123% |
| 119 | M000043_A188001-101_METB_1886.7_EISTR_Galactose, D- (1MEOX) (5TMS) | 11.253 | 1.2353% |
| 66 | EIQTMS_N12C_SD1_2990.6_3150AF12_Galactinol (9TMS); alpha-D-Gal-(1,3)-myo-Inositol (9TMS) | 16.9869 | 1.1924% |
| 63 | EIQTMS_N12C_SD1_2828.4_3223BF31_Gentiobiose (8TMS) | 16.5013 | 1.1271% |
| 112 | M000007_A185001-101_METB_1842.1_EIGTMS_Quinic acid, D(-)- (5TMS) | 11.0428 | 1.1015% |
| 144 | M000633_A189001-101_METB_1894.6_EISTR_Mannose, D- (1MEOX) (5TMS) | 11.4274 | 1.0527% |
| 30 | EIQTMS_N12C_LJALM_1491.9_2236BN40_Malic acid (3TMS) | 8.2398 | 1.0342% |
| 3 | Benzeneacetic acid, ?,4-bis[(trimethylsilyl)oxy]-, trimethylsilyl ester | 12.441 | 0.8522% |
| 159 | Trimethylsilyl 3,4-bis(trimethylsiloxy)cinnamate | 11.9674 | 0.8401% |
| 157 | Silanol, trimethyl-, phosphate (3:1) | 6.3441 | 0.6355% |
| 140 | M000575_A164006-101_MST_1648_EISTR_Tartaric acid (4TMS) | 9.4063 | 0.3857% |
| 24 | EIQTMS_N12C_LJAF_3142.1_2236BN50_[Digalactosylglycerol (9TMS); 880; Melibiose (8TMS); alpha-D-Gal-(1,6)-D-Glc (8TMS)] | 17.4439 | 0.3406% |
| 38 | EIQTMS_N12C_LJARL_1284.6_2236BN14_Glycerol (3TMS) | 6.3223 | 0.2947% |
| 25 | EIQTMS_N12C_LJALD_1326.5_2236BN30_Succinic acid (2TMS) | 6.7727 | 0.2691% |
| 64 | EIQTMS_N12C_SD1_2853.0_3223BF31_Gentiobiose {BP} (8TMS) | 16.638 | 0.2588% |
| 53 | EIQTMS_N12C_SD1_1531.1_3184BF15_4-Aminobutyric acid (3TMS) | 8.6419 | 0.2547% |
| 23 | EIQTMS_N12C_LJAF_2993.6_2236BN50_Galactinol (9TMS); alpha-D-Gal-(1,3)-myo-Inositol (9TMS) | 18.0663 | 0.2032% |
| 82 | M000000_A174001-101_MST_1744.8_EITTMS_ | 10.1784 | 0.1805% |
| 108 | M000000_A282009-101_MST_2828_EIQTMS_ | 16.3417 | 0.1260% |
| 86 | M000000_A179010-101_MST_1792.2_EIQTMS_ | 10.5521 | 0.1146% |
| 29 | EIQTMS_N12C_LJALM_1340.8_2236BN40_Glyceric acid (3TMS) | 6.8504 | 0.1143% |
| 90 | M000000_A210003-101_MST_2099.2_EITTMS_ | 4.9262 | 0.1138% |
| 43 | EIQTMS_N12C_NID1_1424.3_1267BK12_[640; Putrescine (4TMS)] | 8.0677 | 0.1046% |
| 91 | M000000_A211001-101_MST_2105.7_EITTMS_ | 12.6645 | 0.1034% |
| 94 | M000000_A231002-101_MST_2309.5_EITTMS_ | 13.835 | 0.1015% |
| 143 | M000607_A181002-101_METB_1816_EISTR_Shikimic acid (4TMS) | 10.6842 | 0.0991% |
| 69 | EIQTMS_N12C_SD1_3476.1_2009AU04_Melezitose (11TMS) | 19.0841 | 0.0991% |
| 84 | M000000_A177004-101_MST_1770.9_EITTMS | 10.4039 | 0.0978% |
| 12 | EIQTMS_N12C_ATHL_1091.7_3161BN26_L-Alanine (2TMS) | 4.7967 | 0.0975% |
| 16 | EIQTMS_N12C_ATHL_3059.8_3161BN34_[921; Trehalose (8TMS)] | 16.8046 | 0.0951% |
| 132 | M000093_A201001-101_METB_2013.6_EITTMS_Saccharic acid (6TMS) | 12.3897 | 0.0919% |
| 124 | M000055_A193001-101_METB_1927.21519_EIROE_Sorbitol, D- (6TMS) | 11.4916 | 0.0855% |
| 99 | M000000_A252005-101_MST_2527_EIQTMS_ | 14.8072 | 0.0848% |
| 42 | EIQTMS_N12C_NID1_1139.3_1267BK12_[NA] | 5.1641 | 0.0845% |
| 10 | D-Ribose, 2,3,4,5-tetrakis-O-(trimethylsilyl)- | 10.2893 | 0.0822% |
| 56 | EIQTMS_N12C_SD1_1897.9_1267BK12_Glucose methoxyamine (5TMS) | 11.212 | 0.0745% |
| 27 | EIQTMS_N12C_LJALD_2595.6_2236BN30_[Salicylic acid-glucopyranoside (5TMS)] | 16.7796 | 0.0742% |
| 21 | EIQTMS_N12C_LJAF_1912.2_2236BN50_Galactose methoxyamine {BP} (5TMS) | 11.342 | 0.0721% |
| 100 | M000000_A256004-101_MST_2565.3_EIQTMS_ | 15.0472 | 0.0718% |
| 15 | EIQTMS_N12C_ATHL_3013.2_3161BN30_[914; Galactinol (9TMS); alpha-D-Gal-(1,3)-myo-Inositol (9TMS)] | 17.0248 | 0.0655% |
| 31 | EIQTMS_N12C_LJALM_1528.1_2236BN40_Pyroglutamic acid (2TMS) | 8.6388 | 0.0623% |
| 47 | EIQTMS_N12C_NID1_3241.1_1325BK16_[665; 1,4-Anhydrosorbitol (4TMS)] | 18.3619 | 0.0577% |
| 41 | EIQTMS_N12C_MTRR_1209.4_2350BN07_[968; Malonic acid (2TMS)] | 5.7471 | 0.0574% |
| 101 | M000000_A258003-101_MST_2593_EIQTMS_ | 15.3095 | 0.0550% |
| 131 | M000078_A156001-101_METB_1567.1_EIQTMS_Threonic acid (4TMS) | 8.7782 | 0.0499% |
| 22 | EIQTMS_N12C_LJAF_1941.5_2236BN50_Galactitol (6TMS) | 10.947 | 0.0437% |
| 139 | M000571_A158004-101_METB_1572.7_EIMOR_Glutaric acid, 2-oxo- (1MEOX) (2TMS) | 8.9976 | 0.0408% |
| 103 | M000000_A268003-101_MST_2680.9_EIQTMS_ | 17.34 | 0.0403% |
| 60 | EIQTMS_N12C_SD1_2003.4_1286BU10_Gluconic acid (6TMS) | 11.9425 | 0.0398% |
| 32 | EIQTMS_N12C_LJALM_1773.4_2236BN40_[939; 2-Keto-L-gluconic acid (5TMS); A11] | 10.3344 | 0.0387% |
| 109 | M000000_A287007-101_MST_2876.3_EIQTMS_ | 13.1064 | 0.0372% |
| 133 | M000100_A105001-101_CONT-METB_1062.9_EISTR_Lactic acid, DL- (2TMS) | 4.3951 | 0.0372% |
| 98 | M000000_A250002-101_MST_2505.3_EITTMS_ | 14.9203 | 0.0345% |
| 34 | EIQTMS_N12C_LJALM_2409.0_2236BN40_[1-Benzylglucopyranoside] | 14.3419 | 0.0339% |
| 146 | M000671_A274002-101_METB_2743.3_EISTR_Trehalose, alpha,alpha'-, D- (8TMS) | 16.0691 | 0.0321% |
| 117 | M000030_A122001-101_METB_271250_EIBOEL_Valine, DL- (2TMS) | 5.845 | 0.0303% |
| 8 | Cadaverine, N,N,N',N'-tetrakis(trimethylsilyl) | 7.8293 | 0.0291% |
| 59 | EIQTMS_N12C_SD1_1945.7_2081BN04_Coniferyl alcohol (2TMS) | 11.673 | 0.0287% |
| 102 | M000000_A267006-101_MST_2678.6_EIQTMS_ | 15.5348 | 0.0287% |
| 45 | EIQTMS_N12C_NID1_3067.4_1334BV27_[924; Trehalose (8TMS)] | 16.5624 | 0.0285% |
| 36 | EIQTMS_N12C_LJAN_1569.8_2233BN14_Threonic acid (4TMS) | 8.6455 | 0.0278% |
| 95 | M000000_A237002-101_MST_2371.7_EIQTMS_ | 14.1501 | 0.0277% |
| 58 | EIQTMS_N12C_SD1_1936.7_1215BK12_Sorbitol (6TMS); Glucitol (6TMS) | 11.5776 | 0.0276% |
| 107 | M000000_A279002-101_MST_2790.2_EITTMS_ | 17.4009 | 0.0251% |
| 71 | EIQTMS_N12C_SD2_1690.9_1215BK03_Ribose methoxyamine (4TMS) | 9.5224 | 0.0249% |
| 120 | M000044_A264001-101_METB_2647.2_EIQTMS_Sucrose, D- (8TMS) | 19.2427 | 0.0244% |
| 138 | M000517_A106002-101_CONT-MST_1082.2_EISTR_Glycolic acid (2TMS) | 4.5516 | 0.0243% |
| 136 | M000296_A283011-101_METB_2803.034134_EIROE_Gentiobiose (1MEOX) (8TMS) | 16.3267 | 0.0241% |
| 110 | M000000_A354003-101_MST_3537.3_EITTMS_ | 18.0722 | 0.0241% |
| 48 | EIQTMS_N12C_NID1_3334.7_1325BK16_[730; 1,4-Anhydroglucitol (4TMS)] | 17.6792 | 0.0239% |
| 151 | Pentasiloxane, dodecamethyl- | 5.3045 | 0.0229% |
| 149 | M000815_A341001-101_MST_3419.5_EIQTMS_ | 15.8018 | 0.0208% |
| 121 | M000048_A274001-101_METB_2735.4_EISTR_Maltose, D- (1MEOX) (8TMS) | 16.0413 | 0.0207% |
| 118 | M000040_A189002-101_METB_1886.54105_EIROE_Glucose, D- (1MEOX) (5TMS) | 11.2009 | 0.0206% |
| 116 | M000029_A132003-101_METB_1302.3_EISTR_Proline, DL- (2TMS) | 6.653 | 0.0205% |
| 67 | EIQTMS_N12C_SD1_3188.8_1267BK12_4-trans-Caffeoylquinic acid (6TMS) | 17.7694 | 0.0202% |
| 106 | M000000_A277012-101_MST_2777.8_EIQTMS_ | 14.4548 | 0.0200% |
| 150 | N,O,O-Tris(trimethylsilyl)-L-threonine | 7.3494 | 0.0193% |
| 105 | M000000_A277011-101_MST_2775.6_EIQTMS_ | 16.1686 | 0.0192% |
| 93 | M000000_A228001-101_MST_2286.5_EIQTMS_ | 13.673 | 0.0191% |
| 70 | EIQTMS_N12C_SD2_1669.7_1215BK04_Xylose methoxyamine (4TMS) | 9.5778 | 0.0180% |
| 92 | M000000_A214003-101_MST_2140.2_EIQTMS_ | 12.681 | 0.0168% |
| 114 | M000017_A132002-101_METB_1296.3_EISTR_Isoleucine (2TMS) | 6.5597 | 0.0167% |
| 81 | M000000_A171006-101_MST_1706.7_EITTMS_ | 9.7789 | 0.0164% |
| 61 | EIQTMS_N12C_SD1_2012.3_3184BF20_[905; 4-Hydroxy-3-methoxyphenethylene glycol (3TMS)] | 12.042 | 0.0163% |
| 62 | EIQTMS_N12C_SD1_2767.9_1215BK05_Maltose methoxyamine {BP} (8TMS); alpha-D-Glc-(1,4)-D-Glc | 14.5694 | 0.0158% |
| 83 | M000000_A175004-101_MST_1749.6_EITTMS_ | 9.611 | 0.0138% |
| 122 | M000049_A337002-101_METB_3394.2_EIQTMS_Raffinose (11TMS) | 18.4903 | 0.0137% |
| 104 | M000000_A268004-101_MST_2687.9_EIQTMS_ | 15.6203 | 0.0135% |
| 2 | 1,2-Bis(trimethylsiloxy)ethane | 3.7747 | 0.0133% |
| 153 | Silanamine, N,N'-methanetetraylbis[1,1,1-trimethyl- | 3.598 | 0.0132% |
| 141 | M000588_A171012-101_METB_1731.3_EIQTMS_Arabitol, D- (5TMS) | 10.0002 | 0.0131% |
| 89 | M000000_A203005-101_MST_2029.9_EIQTMS_ | 10.4583 | 0.0129% |
| 19 | EIQTMS_N12C_LJAF_1370.6_2236BN50_L-Serine (3TMS) | 7.1256 | 0.0126% |
| 6 | Benzoic acid, 3-methoxy-4-[(trimethylsilyl)oxy]-, trimethylsilyl ester | 10.5013 | 0.0124% |
| 123 | M000054_A150002-101_METB_1505.3_EISTR_Erythritol (4TMS) | 8.2729 | 0.0122% |
| 68 | EIQTMS_N12C_SD1_3406.2_1090DK05_1-trans-Caffeoylquinic acid (6TMS) | 17.9991 | 0.0119% |
| 54 | EIQTMS_N12C_SD1_1631.7_1267BK12_L-Glutamic acid (3TMS) | 9.3229 | 0.0119% |
| 97 | M000000_A239008-101_MST_2395.4_EIQTMS_ | 14.1822 | 0.0117% |
| 52 | EIQTMS_N12C_SD1_1525.1_1267BK12_L-Aspartic acid (3TMS) | 8.5165 | 0.0113% |
| 96 | M000000_A239004-101_MST_2390.6_EIQTMS_ | 13.9786 | 0.0113% |
| 33 | EIQTMS_N12C_LJALM_2188.3_2236BN40_[834; 2-O-Glycerol-beta-D-galactopyranoside (6TMS)] | 13.1448 | 0.0113% |
| 137 | M000364_A127002-101_METB_1253.6_EISTR_Urea (2TMS) | 6.1218 | 0.0111% |
| 74 | EIQTMS_N12C_SD2_2745.6_1267BK12_Maltose methoxyamine (8TMS); alpha-D-Glc-(1,4)-D-Glc | 15.9355 | 0.0107% |
| 5 | Benzoic acid, 3,4-bis[(trimethylsilyl)oxy]-, trimethylsilyl ester | 10.8563 | 0.0107% |
| 125 | M000058_A284001-101_METB_889510_EINS_Maltitol (9TMS) | 16.2386 | 0.0106% |
| 160 | Trimethylsilyl ether of glycerol | 6.4706 | 0.0103% |
| 88 | M000000_A203003-101_MST_2034.8_EIQTMS_ | 12.1478 | 0.0096% |
| 85 | M000000_A177004-101_MST_1771.1_EITTMS_ | 10.8889 | 0.0095% |
| 75 | EIQTMS_N12C_SD2_2768.8_3161BN09_Maltose methoxyamine {BP} (8TMS) | 15.4457 | 0.0090% |
| 51 | EIQTMS_N12C_SD1_1473.3_1215BK22_Citramalic acid (3TMS) | 8.1216 | 0.0085% |
| 156 | Silane, (1-cyclohexen-1-yloxy)trimethyl- | 4.4614 | 0.0083% |
| 78 | L-Tyrosine, N,O-bis(trimethylsilyl)-, trimethylsilyl ester | 11.6756 | 0.0077% |
| 147 | M000720_A240005-101_METB_2402.4_EIMOR_Xylobiose, D- (1MEOX) (6TMS) | 16.3061 | 0.0070% |
| 76 | EIQTMS_N12C_SD2_2993.4_1267BK12_Galactinol (9TMS); alpha-D-Gal-(1,3)-myo-Inositol (9TMS) | 22.3324 | 0.0063% |
| 130 | M000071_A108002-101_MST_1092.9_EISTR_Pyruvic acid (2TMS) | 4.664 | 0.0061% |
| 7 | Butane, 2,3-bis(trimethylsiloxy)- | 4.1427 | 0.0060% |
| 4 | Benzenepropanoic acid, ?,4-bis[(trimethylsilyl)oxy]-, trimethylsilyl ester | 11.407 | 0.0056% |
| 65 | EIQTMS_N12C_SD1_2903.5_2009AU07_Melibiose methoxyamine (8TMS) {BP}; alpha-D-Gal-(1,6)-D-Glc (8TMS) | 14.7756 | 0.0055% |
| 111 | M000000_A381003-101_MST_3814.8_EIQTMS_ | 21.8924 | 0.0055% |
| 135 | M000220_A152003-101_METB_1506.434316_EIROE_Salicylic acid (2TMS) | 8.537 | 0.0052% |
| 55 | EIQTMS_N12C_SD1_1746.6_1215BK07_Fucose methoxyamine {BP} (4TMS) | 10.08 | 0.0052% |
| 128 | M000069_A182004-101_METB-METB_1806.1_EIGTMS_Citric acid (4TMS) | 11.346 | 0.0052% |
| 50 | EIQTMS_N12C_SD1_1359.1_1267BK12_Fumaric acid (2TMS) | 7.0924 | 0.0050% |
| 13 | EIQTMS_N12C_ATHL_1310.2_3161BN21_Glycine (3TMS) | 6.7001 | 0.0045% |
| 79 | M000000_A148003-101_MST_1475.9_EITTMS_ | 8.1232 | 0.0043% |
| 158 | TMS artefact | 4.2992 | 0.0042% |
| 73 | EIQTMS_N12C_SD2_2743.4_3161BN09_Maltose methoxyamine (8TMS) | 20.5802 | 0.0038% |
| 9 | D-Glucose, 2,3,4,5,6-pentakis-O-(trimethylsilyl)- | 11.8053 | 0.0038% |
| 80 | M000000_A159003-101_MST_1585.2_EITTMS_ | 8.9409 | 0.0037% |
| 40 | EIQTMS_N12C_LJARP_2160.4_2236BN20_[849; 1-Methyl-beta-D-galactopyranoside (4TMS)] | 12.834 | 0.0036% |
| 72 | EIQTMS_N12C_SD2_1937.7_2077BN11_Glucuronic acid methoxyamine (5TMS) | 11.5569 | 0.0035% |
| 129 | M000070_A113002-101_METB_1130.4_EIMOR_Oxalic acid (2TMS) | 5.0594 | 0.0035% |
| 87 | M000000_A202004-101_MST_2018.9_EITTMS_ | 12.015 | 0.0030% |
| 44 | EIQTMS_N12C_NID1_3017.4_1267BK12_[910; Galactinol (9TMS); alpha-D-Gal-(1,3)-myo-Inositol (9TMS)] | 22.3334 | 0.0029% |
| 20 | EIQTMS_N12C_LJAF_1635.4_2236BN50_L-Phenylalanine (2TMS) | 9.4649 | 0.0025% |
| 154 | Silanamine, N-[2-[3,4-bis[(trimethylsilyl)oxy]phenyl]ethyl]-1,1,1-trimethyl-N-(trimethylsilyl)- | 10.2531 | 0.0025% |
| 145 | M000657_A194013-101_METB_1940_EIGTMS_Hydrocaffeic acid (3TMS) | 11.7245 | 0.0024% |
| 115 | M000027_A144001-101_METB_1431.3_EISTR_Alanine, beta- (3TMS) | 7.7744 | 0.0023% |
| 26 | EIQTMS_N12C_LJALD_2187.6_2236BN30_[834; 2-O-Glycerol-beta-D-galactopyranoside (6TMS)] | 13.0708 | 0.0023% |
| 148 | M000772_A278010-101_METB_2777.4_EISTR_Cellobiose, D- (8TMS) | 14.4025 | 0.0022% |
| 18 | EIQTMS_N12C_LJAF_1214.4_2236BN50_[953; Malonic acid (2TMS)] | 6.0409 | 0.0021% |
| 11 | EIQTMS_N12C_ATHL_1045.1_3161BN30_Lactic acid (2TMS) | 4.2303 | 0.0017% |
| 35 | EIQTMS_N12C_LJALM_2994.4_2236BN40_Galactinol (9TMS); alpha-D-Gal-(1,3)-myo-Inositol (9TMS) | 16.2123 | 0.0015% |
| 134 | M000102_A143001-101_METB_1428.7_EITTMS_Glutaric acid (2TMS) | 7.6334 | 0.0015% |
| 127 | M000060_A209002-101_METB_2089.1_EITTMS_Inositol, myo- (6TMS) | 11.795 | 0.0013% |
| 28 | EIQTMS_N12C_LJALD_2996.6_2236BN30_Galactinol (9TMS); alpha-D-Gal-(1,3)-myo-Inositol (9TMS) | 17.038 | 0.0010% |
| 57 | EIQTMS_N12C_SD1_1929.5_1215BK09_Mannitol (6TMS) | 11.5378 | 0.0010% |
| 152 | Pyrimidine, 2,4-bis[(trimethylsilyl)oxy]- | 7.0194 | 0.0008% |
| 49 | EIQTMS_N12C_SD1_1162.4_3196BU08_Sulfuric acid (2TMS) | 5.39 | 0.0007% |
